# Investigation into Laser-Vibration-Assisted Cutting of Single-Crystal Silicon by Molecular Dynamics Simulation

**DOI:** 10.3390/mi16121411

**Published:** 2025-12-15

**Authors:** Jianning Chu, Yichen Yang, Yikai Zang, Jinyang Ke, Ziyue Wang, Chen Chen, Jifei He, Aijiang Xu, Zhongdi She

**Affiliations:** 1 Hubei Key Laboratory of Modern Manufacturing Quality Engineering, School of Mechanical Engineering, Hubei University of Technology, Wuhan 430068, China; jianning_chu@163.com; 2 Marine Design and Research Institute of China, Shanghai 200011, China; 3 State Key Laboratory of Intelligent Manufacturing Equipment and Technology, School of Mechanical Science and Engineering, Huazhong University of Science and Technology, Wuhan 430074, China; 4 School of Intelligent Manufacturing, Jianghan University, Wuhan 430056, China; 5 Jiangsu Nuclear Power Corporation, Lianyungang 222044, China

**Keywords:** field-assisted machining, molecular dynamics simulation, single-crystal silicon, crystal defects, material removal mechanism

## Abstract

It is difficult to achieve ultra-precision machining (UPM) on semiconductor materials like single-crystal silicon because of their hardness and brittleness. To solve this issue, numerous field-assisted machining systems and their combinations have been suggested and developed. However, the difficulty in directly observing the physical variables limits our comprehension of the in-depth machining mechanisms of field-assisted machining. In this work, we investigated the machining mechanism of single-crystal silicon under the combination of laser heating and tool vibration using molecular dynamics (MD) simulations. The effect of tool vibration trajectory determined by different tool edge radii is discussed under the condition of raising temperature. The simulation results indicate that the surface morphology is closely related to vibration and heating parameters. Raising the cutting temperature causes a reversed relation between tool edge radius and surface roughness. While the subsurface damage and internal stress are also determined by the tool edge radius and cutting temperature. The findings in this simulation could help to improve the understanding of machining mechanics in multi-field-assisted machining.

## 1. Introduction

Owing to the exceptional optical and electronic properties, single-crystal silicon (Si) is widely employed in Complementary Metal Oxide Semiconductor (CMOS) [1] and photovoltaics [2]. The performance of these devices depends on the machined surface quality of Si components, which necessitates the use of ultra-precision machining (UPM) technologies such as nano-cutting [3], nano-grinding [4], and nano-polishing [5]. However, the primary barriers to achieving high-quality surfaces on single-crystal Si are subsurface damage and unwanted surface defects and cutting tool wear caused by its brittleness and hardness [6,7].

Over the past few decades, various field-assisted machining technologies have been developed to improve the machinability of hard and brittle materials. Laser-assisted machining (LAM) emerged as lasers matured in the 1960s, heating the workpiece with a focused laser spot to thermally soften the workpiece material and drive the removal mode into a ductile regime, thereby reducing the cutting forces and tool wear [8]. In addition to LAM, vibration-assisted machining has been applied in the manufacturing industry since the 1960s [9]. In 1994, Shamoto and Moriwaki proposed elliptical vibration machining (EVM) [10,11] to achieve high-quality surfaces with complex microstructures on brittle materials. By applying high-frequency elliptical vibration to the cutting tool, the cutting motion becomes intermittent, and the material loading is significantly reduced, which enables ductile-regime machining of brittle materials with lower cutting forces and less cutting heat than ordinary cutting [12]. Magnetic field-assisted machining (MFAM), which was first proposed by Yip et al. [13], has been verified as a promising method to achieve UPM of difficult-to-cut materials. Applying a magnetic field is advantageous for improving the plastic-deformation ability of magnetic-conductive materials such as Ti alloys [14]. In recent years, multi-field-assisted machining technologies have been developed to address the bottlenecks of single assistive fields in UPM. Xing et al. [15] combined the EVM with MFAM methods to improve the machining performance of high-entropy alloys. Their results demonstrated that multi-field-assisted machining combines the advantages of single-field machining and effectively enhances the machinability of high-entropy alloys. Ke et al. [16] combined ion implantation with EVM in the machining of sapphire. They used high-energy ions to modify the workpiece surface. Combined with tool vibration, a great enhancement in the machinability of sapphire was achieved. Zhang et al. [17] coupled LAM and EVM to investigate the fabrication process of silicon microlens arrays. Their results suggested that a combination of the single assistive fields broadened the applications of field-assisted machining. In multi-field-assisted machining, the material deformation behavior is more complex than ordinary machining and single-field-assisted machining, as the coupling effect of the assistive fields involves sophisticated interactions between workpieces and tools. Unfortunately, there are few investigations that focus on the machining mechanism during multi-field-assisted machining. And the coupled effect of multiple fields on material removal features and subsurface damage evolution is still unclear.

Based on Newton’s equations of motion, molecular dynamics (MD) simulation has become a viable way to investigate the deformation mechanism of materials in UPM. It has been successfully applied in investigations of LAM [18], EVM [19], and ion-implantation-assisted machining [20]. Dai et al. [21] studied the machining mechanism of single-crystal Si in LAM by employing an intense laser beam to heat the workpiece material. Liu et al. [22] established an MD model to investigate the machining mechanism of reaction-bonded silicon carbide during LAM. They used a uniform initial temperature distribution to explore the effect of laser heating on material deformation and verified the simulation results with experiments. While for EVM, previous simulation results demonstrated that compared to ordinary machining, smaller internal stresses are introduced in the workpiece during machining, which leads to less subsurface damage [23]. Recently, to narrow the gap in parameters between MD simulation and cutting experiments, Liu et al. [24] proposed a new cutting model to simulate the transient cutting process in EVM. They suggested that the dominant removal mechanism of EVM experiences the transition from extrusion to shear in one vibration cycle. Based on this model, they explored the cutting mechanism during the multi-field-assisted cutting that combined LAM and EVM [25]. They analyzed the effect of cutting temperature on the material removal and subsurface damage formation, while the effect of tool vibration trajectory on the machining mechanism under different temperatures was not mentioned. In laser-vibration-assisted machining, tool vibration determines the material removal behavior in a different manner than EVM, since the properties of the workpiece material are significantly influenced by the rising temperature.

Therefore, in the present work, we used MD simulation to investigate the machining mechanism during laser-vibration-assisted machining of single-crystal Si. The material removal behavior and subsurface damage were discussed under different cutting temperatures and tool edge radii, which are important factors that determine the trajectory of the contact point between the tool and the workpiece. The results in this simulation could help to improve the understanding of machining mechanics in multi-field-assisted machining and provide a reference for parameter optimization in the machining process.

## 2. Simulation Method

**Figure 1:**
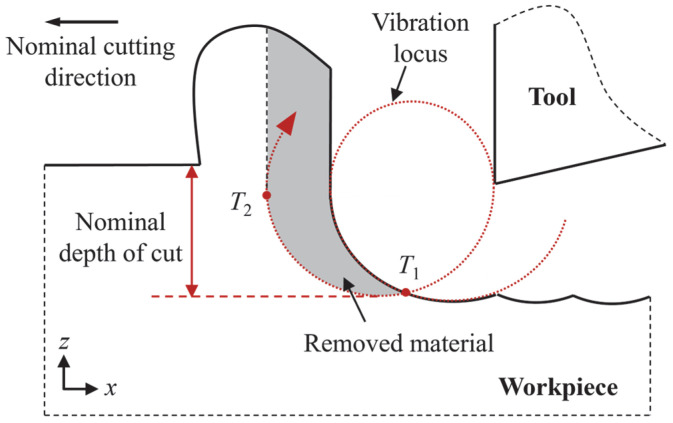
Schematic illustration of EVM. *T*_1_ and *T*_2_ represent the beginning and ending points of the contact stage in one vibration cycle, respectively. Reproduced with permission from C. Liu et al., *Nanoscale Res. Lett.*; published by *Springer Nature*, 2021 [25].

In EVM, the cutting tool moves along the nominal cutting direction and vibrates in the nominal cutting direction and depth of cut direction, as shown in Figure 1. The tool displacement can be calculated by the following:

*x*(*t*) = *A_x_* sin (2*πft*) − *vt* (1)

*z*(*t*) = *A_z_* sin (2*πft* + *φ*) (2)

where *A_x_* and *A_z_* are the vibration amplitudes in the nominal cutting direction and depth of cut direction, respectively. While *f*, *v*, *φ*, and *t* are the vibration frequency, nominal cutting speed, phase shift, and time, respectively.

**Figure 2:**
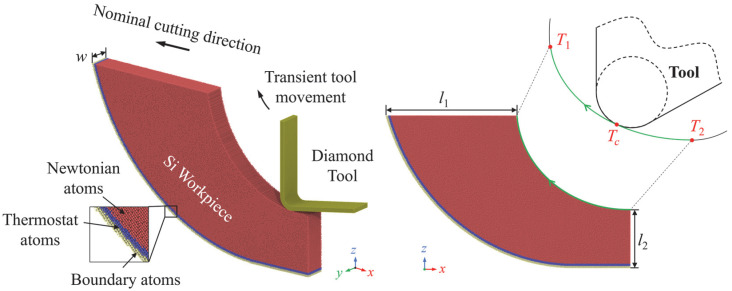
Illustration of the adopted MD cutting model. Reproduced with permission from C. Liu et al., *Nanoscale Res. Lett.*; published by *Springer Nature*, 2021 [25].

The simulation and data processing were performed by LAMMPS [26] and OVITO [27]. Figure 2 displays the adopted cutting model. The single-crystal Si workpiece was shaped according to the trajectory of the tool-workpiece contact point, and the edge of the workpiece was cut to reduce the computational cost [24]. To minimize the computational timesteps, only the contact stage in a vibration cycle (from *T*_1_ to *T*_4_) was simulated. The diamond tool was set as a rigid body while the workpiece was deformable. In accordance with the traditional MD paradigm for UPM, the workpiece atoms are categorized into Newton atoms, thermostat atoms, and boundary atoms [28,29]. The cutting simulation was conducted under different workpiece temperatures and tool edge radii. In previous research, the size of the thermally influenced region in the laser-assisted region is much larger than the deformed region in UPM, which indicates that the variation in temperature in the deformed region is quite small [22]. Therefore, in this research, a unified initial temperature condition was used to simulate the laser heating effect. The atomic interaction between Si-Si and C-C was characterized by ABOP [30], while the interaction between Si-C is described by the Morse potential, which has been shown to be a reliable option with desired calculation efficiency [31,32]. The Morse potential function can be expressed as follows:

(3) $$U_{\mathrm{Si-C}}\;(r_{\mathrm{ij}})\;=\;D_{M}[e^{-2a(r_{\mathrm{ij}\;}-\;r_{M})}-\;2e^{-a(r_{\mathrm{ij}}\;-\;r_{M})}]$$

where *D_M_* represents the cohesion energy, *a* is the modulus of elasticity, and *r_M_* is the equilibrium distance between the atoms. The parameters of the Morse potential for Si-C are set as follows: *D_M_* = 0.435 eV, *a* = 46.487 nm^−1^, *r_M_* = 0.19475 nm [33]. Table 1 lists detailed simulation parameters.

## 3. Results and Discussion

### 3.1. Surface Morphology

**Figure 3:**
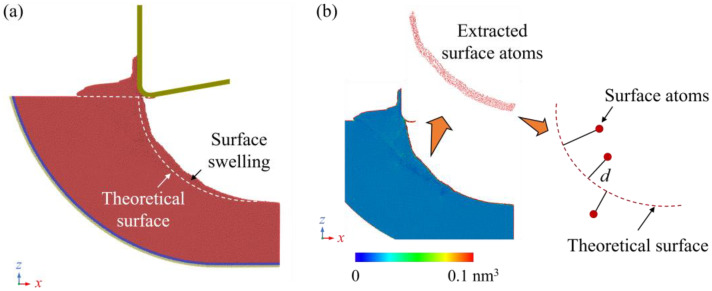
Morphology of the workpiece after cutting. (**a**) Swelling on the machined surface. (**b**) Estimation of surface roughness determined by positions of surface atoms, where the color bar indicates the atomic volume calculated by the Voronoi algorithm.

For single-crystal Si, the machined surface could derive from the theoretical surface due to the surface swelling effect, which is attributed to the strain energy release and phase transition of the metastable phases into the amorphous phase. After machining, the deformed workpiece material tends to return to its balanced position to release the strain energy, and the metastable phases could transform into an amorphous phase with lower density, which could cause obvious expansion of the material. Since the contact state and atomic flow are determined by the material removal thickness and cutting direction, the features of surface swelling in EVM can be significantly different from ordinary machining due to the constant tool vibration. Figure 3a shows a snapshot of the workpiece cut at 300 K by a tool with a 5 nm edge radius. In one vibration cycle, the surface swelling (atoms above the theoretical surface) gradually becomes obvious after the initial contact due to the rise in material removal thickness. As the cutting tool advances, the increase in upward motion of the cutting tool could release the compression and promote the atomic flow of workpiece material. Therefore, surface swelling becomes less obvious at the end of the contact stage.

**Figure 4:**
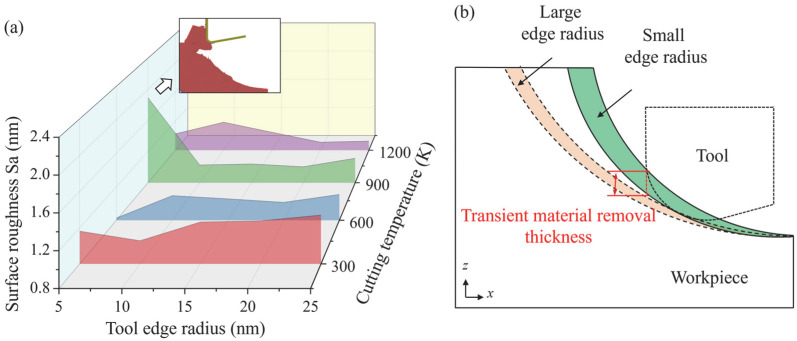
(**a**) The calculated surface roughness under different tool edge radii and cutting temperatures. (**b**) Illustration of the effect of tool edge radius on tool motion.

To evaluate the surface morphology after cutting, the machined surface roughness was calculated based on the position of surface atoms. In MD simulation, the atomic volume determined by the Voronoi algorithm can be used to identify the surface atoms. When atoms are positioned as the cores of a Voronoi cell, the volume of surface atoms in the simulation box can be significantly greater than that of atoms inside the workpiece [34], as shown in Figure 3b. Then, the machined surface roughness (Sa) can be estimated by the following:

(4) $$\mathrm{Sa}\;=\;\frac{1}{A}\iint \left|z(x,\;y)\right|\mathrm{dxdy}\;\approx \;\frac{1}{n}\sum \left|d\right|$$

where *d* is the distance between the position of surface atoms and their theoretical locations, and *n* is the number of surface atoms. Figure 4a shows the calculated machined surface roughness after simulation under different conditions. It is observed that the surface roughness increases with rising tool edge radius when cutting at 300 K and 600 K, while a reversed trend is observed when the temperature increases to 900 K and 1200 K. The remarkably high surface roughness at 900 K and 5 nm tool edge radius is caused by the fracture of the workpiece. During the EVM process (shown in Figure 4b), the transient depth of cut and effective tool rake angle decrease as the tool edge radius increases, causing more surface plowing/rubbing and extrusion removal of workpiece material [24]. Therefore, surface swelling can be more obvious, which tends to increase the surface roughness. When the cutting temperature increases, the plastic deformability of the workpiece is enhanced, and the atomic flow of the surface disordered atoms can be promoted. When a cutting tool with a large edge is used, the tool-workpiece contact area is enlarged, and the surface undulation can be smoothed under high cutting temperature, causing a decrease in surface roughness.

### 3.2. Material Removal Behavior

**Figure 5:**
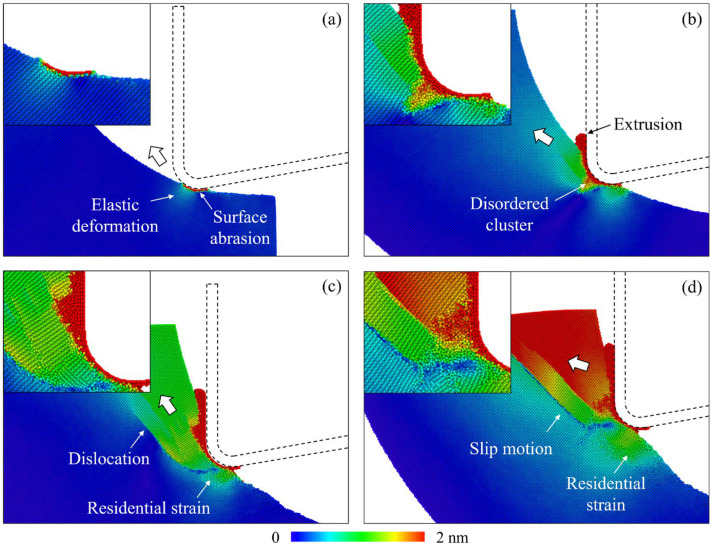
The atomic displacement mapping of workpiece atoms during different cutting stages, where the displacement was calculated over a period of 25 ps. (**a**) 100 ps. (**b**) 200 ps. (**c**) 300 ps. (**d**) 400 ps.

Depending on the cutting conditions, the workpiece material can be removed by extrusion or shear during nanoscale cutting of single-crystal Si [35]. During the EVM process, the material removal mechanism varies continuously due to the continuous changes in the cutting direction and transient material removal thickness. The removal mechanism could transition from extrusion to shear during the workpiece-tool contact stage in a vibration cycle [24]. In MD simulation, the material removal behavior can be identified by analyzing the atomic displacement mapping. When elastic deformation occurs, atomic displacement grows gradually for atoms and their neighbors. An obvious interruption in the displacement magnitude indicates plastic deformation due to the destruction of the crystal structure. Figure 5 shows the relative atomic displacement of different cutting stages in one vibration cycle, which is calculated from a period of 25 ps. In the initial contact stage, nearly no material is removed, and the main workpiece deformation features are surface abrasion and elastic deformation near the contact region, as shown in Figure 5a. As the material removal thickness increases, as shown in Figure 5b, extrusion of the disordered material merges as the dominant removal mechanism, and the disordered atoms are compressed into the subsurface workpiece, forming disordered clusters in the subsurface workpiece. With the advance of the cutting tool, the dislocation path can be observed owing to the increase in transient removal thickness and variation in cutting direction, as shown in Figure 5c. The promoted dislocation propagation and slip motion indicate that the dominant removal mechanism has switched from extrusion to a shear process. The remaining increase in atomic displacement on the machined surface indicates residual strain of the workpiece material. As the slip band extends to the uncut surface, the material is detached from the workpiece, as shown in Figure 5d.

**Figure 6:**
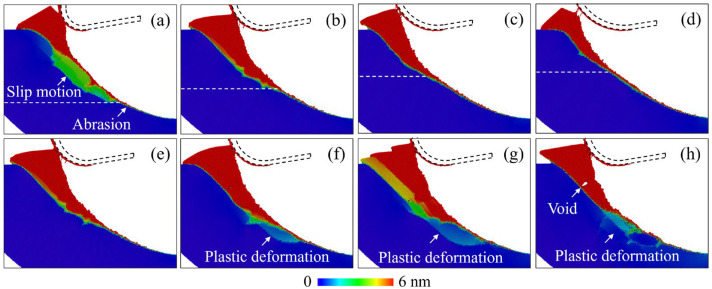
The atomic displacement mapping of workpiece atoms after cutting under different conditions, where the displacement was calculated from the initial position. (**a**–**d**) Cutting with tool edge radii of 10 nm, 15 nm, 20 nm, and 25 nm at 300 K, where the dashed line indicates the approximate position for the beginning of the slip motion. (**e**–**h**) Cutting at 300 K, 600 K, 900 K, and 1200 K with a tool edge radius of 15 nm.

Figure 6 shows the atomic displacement mapping after cutting with tools with different edge radii and cutting temperatures, where the displacement was calculated from the initial position. Referring to Figure 5, an increase in the atomic displacement of the surface atoms indicates surface abrasion, while the slip motion suggests the shear process. When tools with larger edge radii are used, surface abrasion and extrusion removal are promoted since the contact area is enlarged with decreased transient removal thickness, as shown in Figure 6a–d. The plastic deformation mainly concentrates near the surface region, as inapparent atomic displacement was observed in the subsurface workpiece. When the cutting temperature increases, plastic deformation of the subsurface workpiece is promoted as more interruption in the atomic displacement distribution is observed, as shown in Figure 6e–h. In the surface abrasion and extrusion stage, obvious plastic deformation patterns are observed in the subsurface region owing to the enhanced plastic deformability at high temperatures. While in the shear stage, more slip bands are observed with voids formed in the deformed region since rolling and fracture of crystal grains driven by the pulling-up motion from the cutting tool are enhanced at high temperatures [25]. As shown in Figure 6h, the voids generated at high temperature have a minor influence on the quality of the final machined surface since most defects generated in the shear stage can be removed by following the cutting cycle during the EVM process.

### 3.3. Crystal Defects

**Figure 7:**
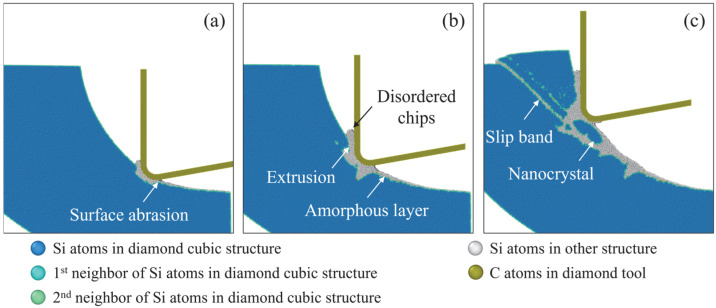
Snapshot of the crystal structure of workpiece atoms in different cutting stages from the simulation at 300 K with a tool edge radius of 5 nm. (**a**) 15 ps. (**b**) 25 ps. (**c**) 35 ps.

The crystal structure of workpiece atoms at various cutting stages from the simulation at 300 K with a tool edge radius of 5 nm is depicted in Figure 7, where the crystal structure was determined using OVITO’s common neighbor analysis (CNA) [36]. In the initial contact stage, the surface abrasion causes the generation of disordered atoms on the contact surface, as shown in Figure 7a. With the increase in material removal thickness, the high-pressure phase transition of the cubic diamond structure to other structures (including metastable phases and amorphous phases) emerges as a significant deformation process. These disordered atoms are extruded into chips ahead of the tool edge, as shown in Figure 7b. Meanwhile, an amorphous layer is left on the machined surface as a result of the surface abrasion and the transition of high-pressure phases into amorphous phases after machining. As the cutting tool further advances (see Figure 7c), the cutting direction changes and material removal thickness increases, facilitating the slip motion along the [111] direction. The dominant material removal mechanism shifts from extrusion to shear, and expansion of disordered atoms into the subsurface workpiece can be observed, creating nanocrystals on the machined surface.

**Figure 8:**
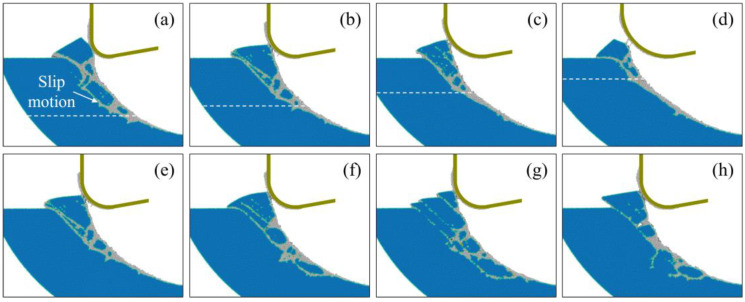
Crystal structure of workpiece atoms after cutting under different conditions. (**a**–**d**) cutting with tool edge radii of 10 nm, 15 nm, 20 nm, and 25 nm at 300 K, where the dashed line indicates the approximate position for the beginning of the slip motion. (**e**–**h**) cutting at 300 K, 600 K, 900 K, and 1200 K with a tool edge radius of 15 nm.

**Figure 9:**
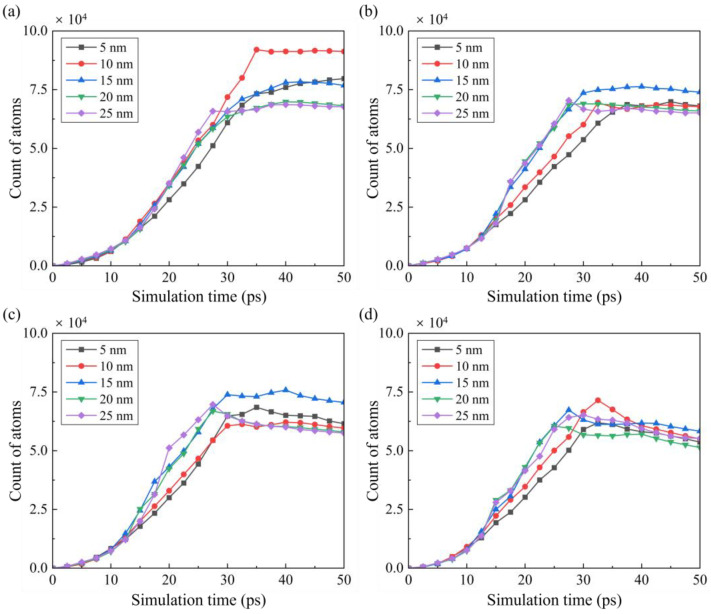
Variation in the disordered atoms cut at (**a**) 300 K, (**b**) 600 K, (**c**) 900 K, and (**d**) 1200 K by tools with different radii.

Figure 8 shows the crystal structure after machining under different conditions of tool edge radius and cutting temperature. During EVM of single-crystal Si, the dominant removal mechanism corresponds to the ratio between the tool edge radius and transient material removal thickness regarding the cutting direction. With increasing tool edge radius, the surface abrasion and extrusion stage is prolonged, forming more disordered atoms on the machined surface. And fewer nanocrystals are generated since slip motion and fracture of crystals are suppressed as the material removal thickness is decreased. As the temperature increases, the amorphous layer becomes less obvious, and more grains are left on the machined surface due to the suppressed high-pressure phase transition and enhanced dislocation-induced plastic deformation at high temperatures. Variation in the disordered atoms during machining under different conditions is shown in Figure 9. During the cutting process, the number of disordered atoms grows sharply from the initial contact stage as the material removal thickness increases. As the cutting tool advances, fewer disordered atoms are generated since the material removal mechanism switches into shear, and the transient material removal thickness decreases [24]. When machining at high temperatures (shown in Figure 9c,d), fewer disordered atoms are generated, and a slight decrease in disordered atoms can be observed in the shear stage, which can be attributed to the recrystallization process [37]. Therefore, the extension of disordered atoms in subsurface workpieces can be narrow and discontinuous under higher temperatures, as shown in Figure 9.

**Figure 10:**
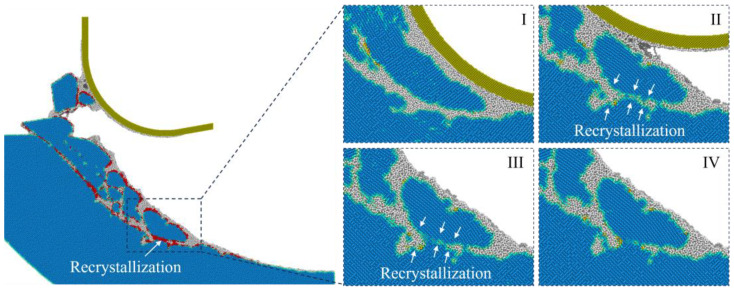
Illustration of the recrystallization during cutting at 1200 K with a tool edge radius of 25 nm, where the recrystallized atoms are in red color. (**I**–**IV**) shows the snapshots of the deformed region during different cutting stages.

**Figure 11:**
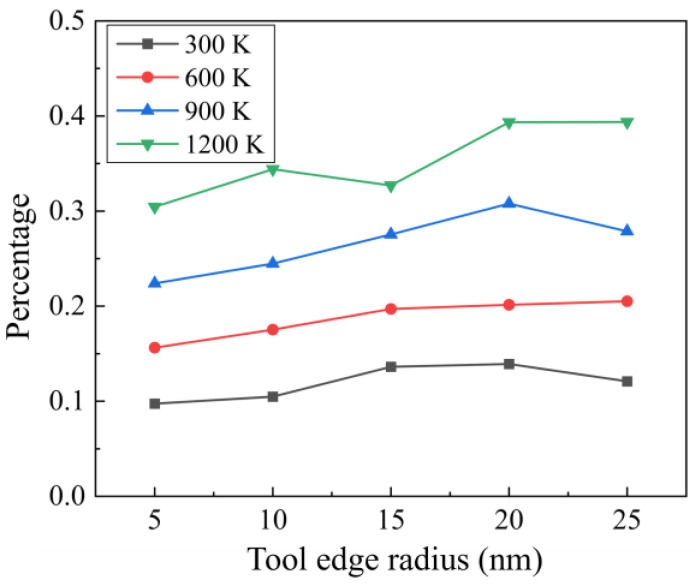
The percentage of recrystallization under different cutting conditions.

For materials such as single-crystal Si, recrystallization of disordered phases into crystal structures can be observed during high-temperature machining [38]. Figure 10 shows the recrystallization process when cutting at 1200 K with a tool edge radius of 25 nm, where the recrystallized atoms were identified by the CNA. It is observed that the recrystallization process is most obvious in the initial stage after machining, where the compressive stress remains in the deformed region since the transition of disordered Si into a cubic diamond structure can be promoted by applying compression and appropriate temperature [39]. Figure 11 shows the percentage of recrystallization under different cutting conditions, which is defined as the ratio between recrystallized atoms and the total disordered atoms. It is observed that recrystallization is promoted by raising the cutting temperature and tool edge radius. For blunt tools, a smaller material removal thickness indicates less generation of disordered atoms, while the enlarged contact area facilitates compression of the workpiece material, which is responsible for the promoted recrystallization percentage.

### 3.4. Stress Analysis

**Figure 12:**
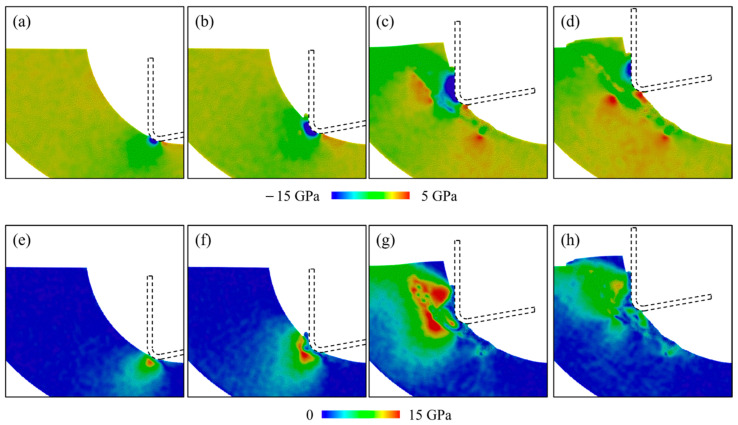
Distribution of the (**a**–**d**) hydrostatic stress and (**e**–**h**) von Mises stress in different cutting stages.

To further investigate the formation mechanism of subsurface damage, the internal stress in the workpiece was analyzed. The hydrostatic stress and von Mises stress in the workpiece were calculated based on the stress tensors from LAMMPS [40]:

(5) $$\sigma_{\mathrm{hydrostatic}\;}=\;\frac{1}{3}{(\sigma }_{\mathrm{xx}}+\;\sigma_{\mathrm{yy}}+\;\sigma_{\mathrm{zz}})\;$$

(6) $$\sigma_{\mathrm{von}\;\mathrm{Mises}}\;=\sqrt{\frac{1}{2}((\sigma_{\mathrm{xx}}\;-\;\sigma_{\mathrm{yy}}{)}^{2}+(\sigma_{\mathrm{yy}}\;-\;\sigma_{\mathrm{zz}}{)}^{2}+(\sigma_{\mathrm{zz}}-\sigma_{\mathrm{xx}}{)}^{2}+6(\tau_{\mathrm{xy}}^{2}+\tau_{\mathrm{yz}}^{2}+\tau_{\mathrm{zx}}^{2}))\;}$$

where *σ_x__x_*, *σ_y__y_*, *σ_z__z_*, *τ_xy_*, *τ_xz_*, and *τ_yz_* are stress tensors. In this simulation, we used a spatial average of a cubic box of 2 nm to average the stress tensor, aiming to eliminate the fluctuation in the numerical calculation. Figure 12 shows the distribution of hydrostatic stress and von Mises stress in different cutting stages, where the workpiece was cut at 300 K by a tool with a 5 nm radius. Negative hydrostatic stress indicates compression, while positive hydrostatic stress suggests tension. In the initial contact stage, a high compressive zone is formed near the tool edge, causing surface abrasion and extrusion by introducing the high-pressure phase transition. A slight increase in tensile stress is observed behind the contact region due to the tearing of the workpiece by the cutting tool motion. As the material removal thickness increases, the compressive region is enlarged, and a shear region is formed due to the extrusion of disordered atoms. When the cutting process comes into the shear stage, variation in the cutting direction promotes the pulling-up motion, which contributes to the increase in tensile stress behind the contact region and in the subsurface workpiece. At the end of cutting, the internal stress decreases due to the detachment of crystal grains.

**Figure 13:**
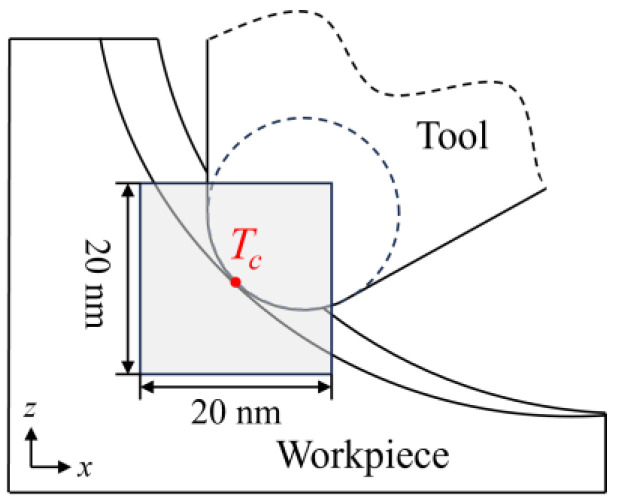
Definition of the deformed region for average stress calculation.

**Figure 14:**
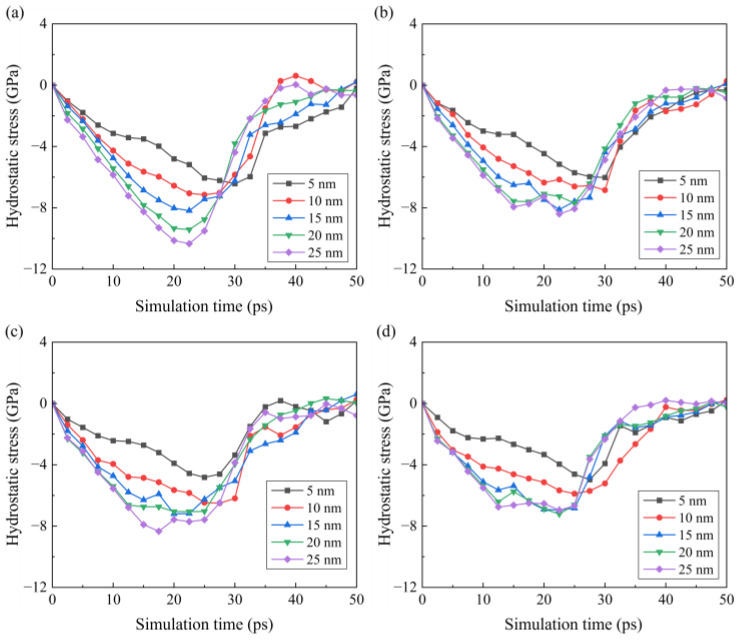
Variation in the hydrostatic stress and in the workpiece under different temperatures: (**a**) 300 K, (**b**) 600 K, (**c**) 900 K, and (**d**) 1200 K.

**Figure 15:**
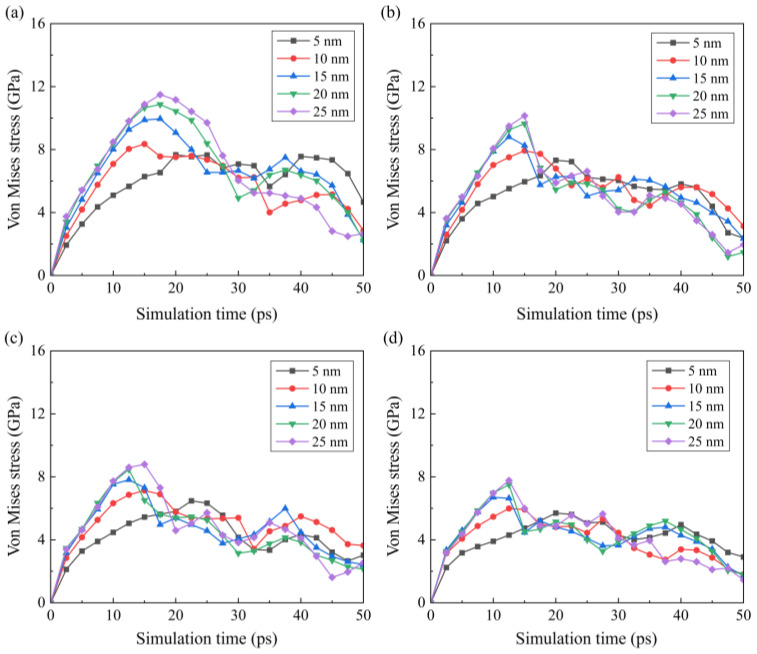
Variation in the von Mises stress and in the workpiece under different temperatures: (**a**) 300 K, (**b**) 600 K, (**c**) 900 K, and (**d**) 1200 K.

To investigate the variation in internal stress during the cutting stage, the average stress in a deformed region during the cutting stage under different conditions is calculated. The deformed region is defined as a box of 20 nm centered at *T_c_*, as shown in Figure 13. The calculated average hydrostatic stress and von Mises stress are shown in Figure 14 and Figure 15. During the cutting process, the hydrostatic stress increases as the material removal thickness increases from the initial contact stage. As the pulling-up motion increases and the detachment of crystal grains increases, the compressive stress decreases at the later cutting stage. Similar trends can be observed for the von Mises stress shown in Figure 15. As the cutting temperature increases, both hydrostatic stress and von Mises stress show an apparent decrease owing to the thermal softening of the workpiece material. For a workpiece machined by a blunt tool, an increase in internal stress is observed as the contact area is enlarged. In addition, when the tool edge radius increases, the internal stress reaches its maximum earlier, which is attributed to the fact that the detachment of the deformed region is promoted by a blunt tool. While this phenomenon becomes less apparent when the cutting temperature increases, since the enhanced plastic deformability at high temperature facilitates the slip motion and detachment of the crystal grains.

## 4. Conclusions

In this research, the material removal mechanism and subsurface damage generation in laser-vibration-assisted machining of single-crystal Si were examined using MD simulation. The impact of cutting temperature and tool edge radius on the machining mechanism was discussed. The following is a summary of the key findings:

(1) In one vibration cycle, surface swelling gradually becomes obvious after the initial contact due to the rise in material removal thickness, while it becomes less apparent at the end of the contact stage. When cutting at room temperature, the surface swelling is more obvious, and the surface roughness is increased when tools with a large edge radius are adopted. As the cutting temperature increases, a decrease in surface roughness is observed when the tool edge radius is increased.
(2) As the tool edge radius increases, more surface abrasion and extrusion removal can be observed during the cutting stage. As the cutting temperature increases, the plastic deformation in the subsurface workpiece is promoted. In the surface abrasion and extrusion stage, obvious plastic deformation patterns are observed. During the shear stage, more slip bands are observed with voids formed in the subsurface workpiece.
(3) With increasing tool edge radius, more disordered atoms are generated on the machined surface since the surface abrasion and extrusion stage is prolonged. As the temperature increases, the amorphous layer becomes inapparent, and more grains are left on the machined surface, which can be attributed to the enhanced dislocation-induced plastic deformation. Furthermore, recrystallization is promoted by raising the cutting temperature and tool edge radius.
(4) As the cutting temperature increases, both hydrostatic stress and von Mises stress show an apparent decrease owing to the thermal softening of the workpiece material. For workpieces machined by a blunt tool, an increase in internal stress is observed, and the internal stress reaches a maximum earlier. While this phenomenon becomes less apparent when the cutting temperature increases, since the enhanced plastic deformability facilitates the detachment of the crystal grains.

## Figures and Tables

**Table 1:** Parameters of the MD model.

Parameters	Value
Size of workpiece (*l*_1_*/l*_2_*/w*)	50 nm/15 nm/8.7 nm
Crystal orientation	*x*:(001)[100]; *y*:(001)[010] (workpiece and tool)
Total atoms	Approximately 1.9–2.1 million
Cutting temperature	300 K, 600 K, 900 K, 1200 K
Vibration frequency (*f*)	500 MHz
Phase difference (*φ*)	90°
Vibration amplitude (*A_x_*/*A_d_*)	40 nm/40 nm
Nominal depth of cut	40 nm
Tool edge radius	5 nm, 10 nm, 15 nm, 20 nm, 25 nm
Nominal cutting speed	3 m/s
Timestep	1 fs

## Data Availability

The original contributions presented in the study are included in the article; further inquiries can be directed to the corresponding author.
